# Use of industry 4.0 technologies to reduce and valorize seafood waste and by-products: A narrative review on current knowledge

**DOI:** 10.1016/j.crfs.2023.100505

**Published:** 2023-04-20

**Authors:** Abdo Hassoun, Janna Cropotova, Hana Trollman, Sandeep Jagtap, Guillermo Garcia-Garcia, Carlos Parra-López, Nilesh Nirmal, Fatih Özogul, Zuhaib Bhat, Abderrahmane Aït-Kaddour, Gioacchino Bono

**Affiliations:** a Univ. Littoral Côte D’Opale, UMRt 1158 BioEcoAgro, USC ANSES, INRAe, Univ. Artois, Univ. Lille, Univ. Picardie Jules Verne, Univ. Liège, Junia, F-62200, Boulogne-sur-Mer, France; b Sustainable AgriFoodtech Innovation & Research (SAFIR), Arras, France; c Department of Biological Sciences, Ålesund, Norwegian University of Science and Technology, Larsgårdsvegen 4, 6025, Ålesund, Norway; d School of Business, University of Leicester, Leicester, LE2 1RQ, UK; e Sustainable Manufacturing Systems Centre, School of Aerospace, Transport & Manufacturing, Cranfield University, Cranfield, MK43 0AL, UK; f Department of Agrifood System Economics, Centre ‘Camino de Purchil’, Institute of Agricultural and Fisheries Research and Training (IFAPA), P.O. Box 2027, 18080, Granada, Spain; g Institute of Nutrition, Mahidol University, 999 Phutthamonthon 4 Road, Salaya, Phutthamonthon, Nakhon Pathom, 73170, Thailand; h Department of Seafood Processing Technology, Faculty of Fisheries, Cukurova University, 01330, Balcali, Adana, Turkey; i Division of Livestock Products Technology, SKUAST-Jammu, Jammu, 181102, J&K, India; j Université Clermont Auvergne, INRAE, VetAgro Sup, UMRF, Lempdes, France; k Institute for Biological Resources and Marine Biotechnologies, National Research Council (IRBIM-CNR), Mazara Del Vallo, Italy; l Dipartimento di Scienze e Technologie Biologiche, Chimiche e Farmaceutiche (STEBICEF), Università Di Palermo, Viale Delle Scienze, 90128, Palermo, Italy

**Keywords:** Seafood processing by-product, Fish side stream, Fourth industrial revolution, Artificial intelligence, Big data, Smart sensors, Internet of things, Sustainability, Valorization, Innovative technologies, Circular economy

## Abstract

Fish and other seafood products represent a valuable source of many nutrients and micronutrients for the human diet and contribute significantly to global food security. However, considerable amounts of seafood waste and by-products are generated along the seafood value and supply chain, from the sea to the consumer table, causing severe environmental damage and significant economic loss. Therefore, innovative solutions and alternative approaches are urgently needed to ensure a better management of seafood discards and mitigate their economic and environmental burdens. The use of emerging technologies, including the fourth industrial revolution (Industry 4.0) innovations (such as Artificial Intelligence, Big Data, smart sensors, and the Internet of Things, and other advanced technologies) to reduce and valorize seafood waste and by-products could be a promising strategy to enhance blue economy and food sustainability around the globe. This narrative review focuses on the issues and risks associated with the underutilization of waste and by-products resulting from fisheries and other seafood industries. Particularly, recent technological advances and digital tools being harnessed for the prevention and valorization of these natural invaluable resources are highlighted.

## Highlights

• Food waste is one of the major challenges facing many agri-food systems including the seafood sector.
• ArTificial Intelligence, and other Industry 4.0 technologies are increasingly leveraged in various food-related sectors.
• Advanced technological solutions proposed for a more sustainable utilization of seafood side-streams.
• Industry 4.0 technologies have a huge potential to reduce and valorize seafood waste and by-products.

## Graphical abstract

**Figure:**
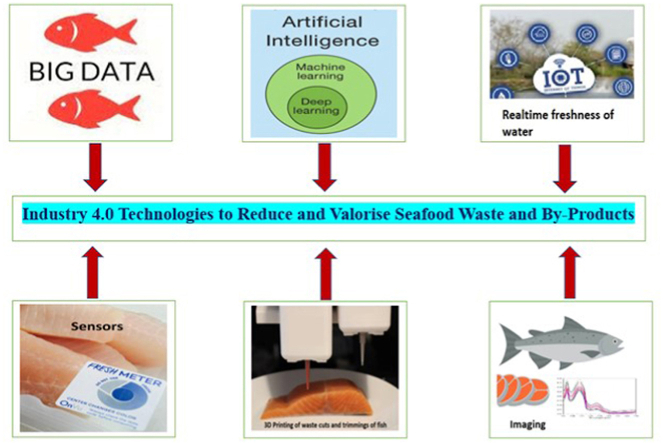

## 1 Introduction

Seafood from fisheries and aquaculture, play a pivotal role in human diet and health and contribute significantly to global food nutritional security, but considerable amounts of these valuable resources are lost or wasted during fishing, harvest, transportation, processing, and distribution before reaching the consumer table (Roobab et al., 2022; Venugopal 2021). Seafood discards and by-products could be used in different food and biotechnological applications (Karkal and Kudre 2020, 2022; Nag et al., 2022; Shahidi et al., 2019). Underutilized fish parts, including head, fins, scales, bones, viscera, trimmings, skin, and other fish discards could be valuable sources of edible proteins and other nitrogenous compounds (such as peptides, amino acids, and hydrolysates), chitin and other polysaccharides, lipids, vitamins, carotenoids, among other bioactive compounds (Hassoun et al. 2021; Ozogul et al., 2021; Venugopal 2022). However, their full potential has not yet been realized due to several obstacles, such as high perishability, consumer acceptance of food waste and rest raw materials and their derivative products, as well as lack of adequate infrastructure (Han et al., 2021; Hassoun et al., 2022a, Hassoun et al., 2022b, Hassoun et al., 2022c, Hassoun et al., 2022d, Hassoun et al., 2022e, Hassoun et al., 2022f, Hassoun et al., 2022g; Kang et al., 2022).

Recent global challenges, such as climate change, increasing global population, overfishing and depletion of wild fish stocks, and marine ecosystem destruction have left humans no choice but to innovate and investigate further to develop new methods and more efficient strategies and seek alternative solutions that could help reduce the generation of seafood waste and by-products and exploit them in the best possible manner. To this end, digitalization and Industry 4.0 technologies have gained enormous attention in recent years to improve efficiencies and productivity, enhance sustainability, and increase food quality, safety, and traceability (Hassoun et al., 2023; Hassoun, Abdullah, Aït-kaddour, Ayşegül Beşir, et al. 2022; Hassoun, Aït-kaddour, Abu-mahfouz, Rathod, et al., 2022; Hassoun et al., 2022a, Hassoun et al., 2022b, Hassoun et al., 2022c, Hassoun et al., 2022d, Hassoun et al., 2022e, Hassoun et al., 2022f, Hassoun et al., 2022g; Hassoun et al., 2022a, Hassoun et al., 2022b, Hassoun et al., 2022c, Hassoun et al., 2022d, Hassoun et al., 2022e, Hassoun et al., 2022f, Hassoun et al., 2022g). In the seafood sector, digital and Industry 4.0 technologies, such as Artificial Intelligence (AI), Big Data (BD), the Internet of Things (IoT), blockchain, 3D printing, and robotics could help to meet expansion needs of the fisheries and aquaculture industries (Hassoun et al., 2022; Hjellnes et al. 2020; Rowan 2022). Beside the aforementioned technologies, the increasing access to information and communications for everyone has opened up new opportunities through digitalization and the application of digital tools, which are based on Industry 4.0 concepts (Annosi et al., 2021; UNEP DTU Partnership and United Nations Environment and Programme 2021). Digital platforms and digital technologies are becoming increasingly applied in the fight against food waste (Benyam et al. 2021; Cane and Parra 2020).

The role of Industry 4.0 technologies in promoting circular economy to achieve a transition to net zero through efficient waste management was recently highlighted (Agustiono et al., 2023). The implication of these advanced technologies in the reduction of wastes in water, energy, and food was also discussed (David et al., 2022). Recent technological advances used for reducing food losses in the supply chain of fresh agricultural products, such as fruits and vegetables were recently reviewed by Onwude et al. (2020). However, to the best of our knowledge, a limited number of reviews exists in the literature to explore the role of digital, Industry 4.0, and other emerging technologies in the context of seafood loss and waste. Therefore, this review focuses on recent publications dealing with emerging and innovative strategies applied for the prevention and valorization of marine wastes and by-products, focusing mainly on Industry 4.0 technologies.

## 2 Seafood waste and by-products

By 2050, the world population is expected to grow by about one-third, raising the global need for alternative sustainable and renewable sources of high-quality protein able to satisfy the growing demand for food, nutraceuticals, and pharmaceuticals, which fulfil the concepts of sustainable circular economy. This requires finding additional or alternative sources of high-quality protein that can be simultaneously safe and acceptable to the consumer, in addition to exploit the available resources in the best and most efficient way (Venugopal and Sasidharan 2022; Visconti et al., 2020).

Seafood products are valuable marine resources rich in high-quality protein and other essential nutrients. The amount of protein in seafood muscle/flesh is ranging from 18 to 23% depending on the nature of a seafood product (fish, shellfish, or cephalopods), as well as their habitats (ocean/coastal, or fresh waters) (Venugopal 2009). Moreover, not only seafood flesh used for direct human consumption is rich in high-quality protein, but also the other parts of seafood used indirectly. A number of studies have shown that valuable bioactive proteins are found in appreciable amounts in seafood side streams and by-products such as viscera, liver, kidney, eggs, head, backbone, and skin (Ananey-Obiri et al. 2019; Coppola et al., 2021; Mutalipassi et al., 2021; Nirmal et al., 2022a).

Global fish production is expected to reach 200 metric tons (MT) by 2029. This represents a 14% increase (25 MT) compared to the pre-COVID period of 2017–2019 (OECD/FAO 2022). In addition, the global consumption of seafood is expected to increase noticeably over the next decade, at a quicker rate than meat consumption. Now, global per capita consumption of seafood products equals to 20.1 kg, covering around 20% of the total average intake of animal protein. Thus, to meet the continuously rising demand for proteins by 2050, aquaculture production alone would need to achieve 129 MT according to status quo consumption (Boyd et al. 2022; Peñarubia 2021). However, in the fish processing industry, only 30–40% of the fish raw material are processed into high quality products (fish steaks, medallions, or fillets) for human consumption. Thus, a huge amount of rest raw material (from 25% to 70%) including fins, heads, skin, and viscera are discarded or used for low-value applications such as fish meal and oil, or animal feed (Peñarubia 2021). Annually, 15 million tons of fish catch are used to produce those products (Olsen et al. 2014). However, if the quality of by-catches or side streams does not correspond to the quality requirements for these categories of products, the residual material often finds its way in the production of biogas, compost, or even low-value options such as incineration or landfilling (Venugopal 2021; Venugopal and Sasidharan 2022). The EU Waste Framework Directive sets a hierarchy of the best possible options for waste valorization practices (Directive, 2006/12/EC). Despite the low value traditionally assigned to fishery by-products, from the huge mass of unused/underutilized resources, a significant number of bioactive compounds could be extracted and valorized, including protein ingredients. Thus, leaving aside the principle of prevention and reductions, the most efficient and profitable option for reducing seafood waste is the extraction and production of high-added value products and ingredients (such as enzymes, bioactive peptides, protein hydrolysates, collagen and gelatin peptides, lipids rich in long-chain polyunsaturated fatty acids, and chitin and chitosan) (Venugopal 2009). Annually, discards from the world's fisheries exceed 9 million tons equivalent to about 10% of the annual catch (FAO 2020). In addition, a huge amount of loss and waste occurs across the fish value chain including “non-target” fish species, seafood processing residues and by-products having no or low commercial value due to undesirable characteristics (Hassoun et al., 2021; Ozogul et al., 2021).

Global fish discards and residual biomass are generated at different points in the value chain as by-catch, on-board handling, landing centers, transportation, storage, retailers, and consumers. Since the beginning of the 21st century, the European Parliament and the Council of the European Union have been alerted about the high residual volumes generated with the practice of “discard at sea” of unintentional catches (Olsen et al., 2014). In fish capture, a considerable portion of marine catch is usually dumped back into the sea due to various reasons. These include sizes of species, non-target species, or non-marketable species that are damaged or dead. This method is considered environmentally unsustainable from the biogeochemical point of view, since these biomasses are generating huge organic matter intake in the seawater.

In 2013, to prevent ecological and economical losses, the third version of the Common Fisheries Policy, including the landing obligation EU directives, was adopted by the European Parliament and the European Council. As of 2019, the Landing Obligation of the European Commission Common Fisheries Policy forced all fishing vessels to keep and not discard all the species which are subjected to quota or have a minimum legal size, as well as underutilized commercial species (Uhlmann et al. 2019).

The fishing industry is currently focused on getting the most effective value out of primary products, with little interest in side streams and by-products, resulting in lost economic opportunities due to lack of investment in advanced technologies. Considering the future scenario of food scarcity by 2050 (Venugopal and Sasidharan 2022), there is a strong demand for new technical and organizational solutions aimed to offer better exploitation of seafood side stream and maximize their economic potential. In this regard, Industry 4.0 may offer advanced technological solutions for a more sustainable utilization of seafood side-streams (Hassoun, Aït-kaddour, Abu-mahfouz, Rathod, et al., 2022). The smart and sustainable valorization of seafood residuals obtained from by-catches and fish processing activities is a key route for future seafood field from not only the environmental point of view, but also economic value that can be obtained from these side-streams.

## 3 Common strategies used for reduction and valorization of seafood waste

The utilization of seafood by-products and side streams as a source of proteins, polysaccharides, lipids, enzymes, pigments, and minerals has become increasingly popular for various industrial applications (Nirmal and Maqsood 2022). Fish processing waste and by-products contain higher protein concentration (58%) (Nirmal et al., 2022b), whereas shellfish processing waste has a higher concentration of polysaccharides (20–46%) and minerals (30–60%) (Nirmal et al., 2020).

**Fig. 1:**
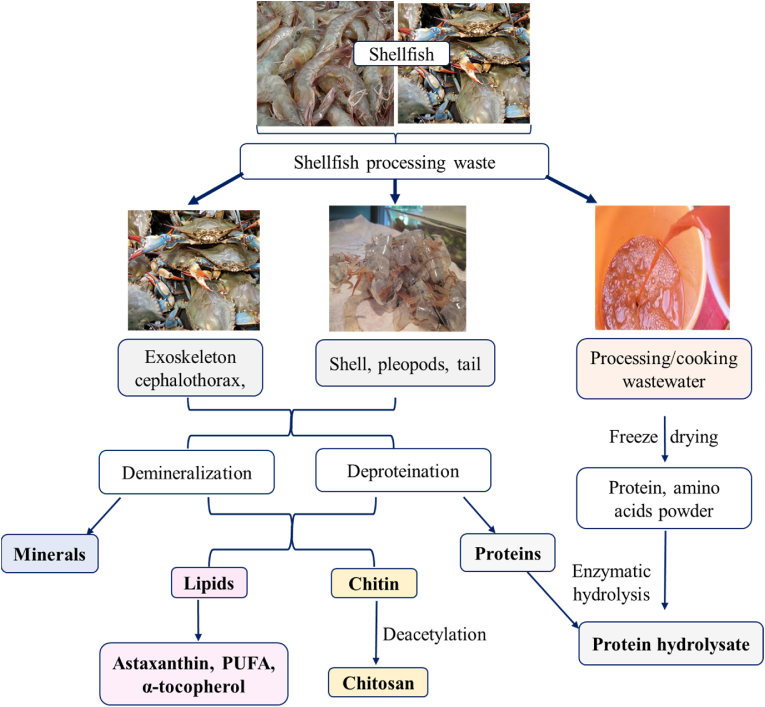
Schematic presentation of shellfish process material utilization for value added products (Nirmal et al., 2020).

The traditional methods of extracting and processing seafood waste and by-products involve chemical treatment with strong alkalis and acids, which cause a loss of functionality in the end-product, environmental pollution, and health hazards for operators (Gao et al., 2021). As a result, alternative methods based on enzyme and microbial fermentation are getting more attention due to their environmental friendliness, controlled process, and high-quality end products (Abuine et al. 2019). Recently, non-thermal technologies have also been used for bioactive compound extraction from seafood residuals (Ali et al., 2021). While these non-thermal technologies have the potential to enhance the extraction process, improve waste utilization, and reduce environmental impact, their applications are still in the early stages of development and further research and studies are needed for successful scale-up to industrial level. A combined approach of various non-thermal technologies along with enzyme hydrolysis process seems to be the fastest and most cost-effective and eco-friendly strategy for seafood waste valorization. Enzymatic hydrolysis process involves the application of endogenous or exogenous enzyme from different sources, such as plants (papain, ficin, etc.), animals (pepsin, trypsin, etc.), and microorganisms (e.g., protease) (Nirmal et al., 2022). Fig. 1 illustrates the extraction of bioactive compounds from the shellfish processing industry.

Enzymatic hydrolysis starts with selecting proper enzyme or combination of enzymes, depending on the end-product requirement. Then, the selected enzyme-to-seafood rest raw material ratio, optimum pH, and temperature conditions are employed to obtain the required degree of hydrolysis. Finally, the reaction is terminated when the desired hydrolysis achieved (Fan et al., 2019; Gao et al., 2021). These processes can be controlled or modulated according to the required final product quantity, functionality, and bioactivity. However, enzyme stability and the cost of the purified enzyme remain the major two obstacles for the scale-up process (Gui et al., 2022).

The fermentation process uses seafood side streams and by-products as a substrate for the growth of inoculated microorganisms. These microorganisms can degrade biopolymers (proteins and polysaccharides) into smaller particles by secreting protease and hydrolase enzymes. Commonly used microorganisms are *Aspergillus oryzae, Streptococcus thermophiles, Saccharomyces cerevisiae*, and lactic acid bacteria (Mao et al., 2017). Additionally, the microbial fermentation process is known to neutralize antinutritional and allergic compounds in the reaction mixture (Raveschot et al., 2018). The main problems with the fermentation process are cross contamination with other microorganisms, strict fermentation conditions, and expensive down steam process (Hou et al., 2017). Therefore, taking into consideration the pros and cons of present techniques, a combined approach of non-thermal techniques with enzyme hydrolysis could be considered as efficient in reducing time, energy, and solvent, achieving higher extraction yields (Ali et al., 2021).

High hydrostatic pressure (HHP) in combination with enzymatic hydrolysis was applied for the extraction of protein hydrolysates from tilapia processing waste (Hemker et al., 2020). HHP enhanced the protein solubility and enzyme activity thereby reducing the extraction time and producing higher yield with high antioxidant activity of the protein hydrolysate. Gulzar and Benjakul (2020) extracted lipid from the cephalothoraxes of Pacific white shrimp (*Litopenaeus vannamei*) using ultrasound-assisted process (UAE). Before the UAE extraction, they pre-treated cephalothoraxes with pulse electric field to enhance the lipid extraction by degrading the shell structure. The process recovery provided higher yields with high polyunsaturated fatty acids and carotenoid content (Gulzar and Benjakul 2020). Hence, the literature suggests that seafood side streams and by-products can be valorized using the aforementioned technologies to obtain high-value bioactive compounds with various functional properties, which can be applied as nutraceuticals, functional food ingredients, and for disease prevention (Nirmal et al., 2022; Nirmal and Maqsood 2022; Nirmal et al. 2023).

## 4 Industry 4.0 technologies and digital tools

**Fig. 2:**
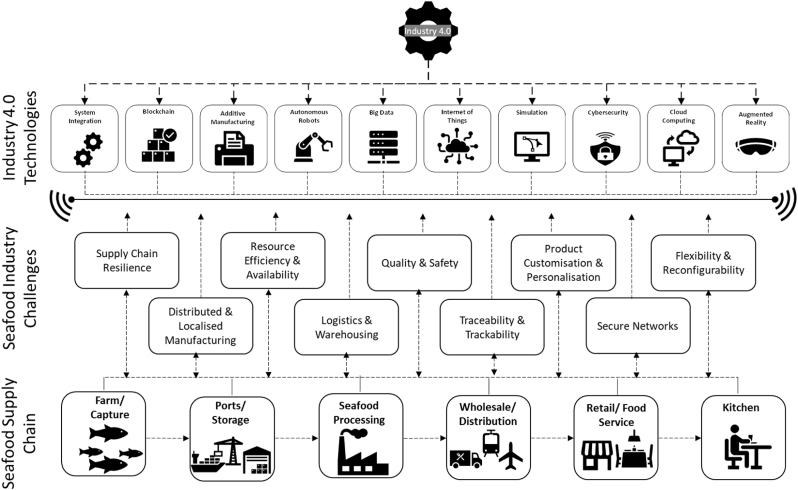
The relationship between Industry 4.0 technologies and the seafood supply chain in addressing the challenges faced by the seafood industry (Bader and Rahimifard 2018; Garrett et al. 2019).

The fourth industrial revolution (Industry 4.0) technologies have the potential to revolutionize and improve the circularity of resources within the seafood supply chain while also tackling the numerous challenges facing the seafood industry. Fig. 2 provides examples of Industry 4.0 technologies, such as system integration, blockchain, additive manufacturing, autonomous robots, AI, BD, IoT and smart sensors, simulation, cybersecurity, cloud computing, and augmented reality (Jagtap et al., 2021a, Jagtap et al., 2021b), which can increase circularity within seafood supply chains and, as a result, reduce loss and waste or provide solutions for waste valorization.

In addition to Industry 4.0 paradigm, another related concept that has received enormous attention in recent years is digitalization and the use of digital tools. Digital technologies can refer to a set of tools, e.g., BD, blockchain, AI, IoT, and cloud computing that are used to facilitate connectivity and information sharing (Abbate et al. 2023; Annosi et al., 2021). These technologies hold significant potential to increase efficiency and value added in food supply chains, thus decreasing food waste. For example, instead of traditional labels (“use-by” or “best-before” dates), smart sensors and digital solutions could be implemented to provide real-time expiration dates (Nayyar et al. 2018; Tichoniuk et al. 2021).

With the development of digital platform technologies, smart food sharing technologies have become increasingly popular as a means for reducing food waste. Many waste apps (software applications), designed to run on mobile devices (e.g., phones, tablets, and watches) have emerged in recent years to minimize food waste by linking manufacturers, supermarkets, cafes, restaurants, and individual households to local communities. Examples of such Apps are “Too Good To Go”, “OLIO”, and “Myfoody” (Cane and Parra 2020; Ciccullo et al., 2022; Crew 2021; X. Liu et al., 2022a, Liu et al., 2022b).

Apart their use in food waste reduction and valorization, various digital technologies and tools have been developed to manage waste in different other industries, including transport, energy, pharmaceutical industry, health, bioeconomy, tourism and culture, among others (Lekkas et al. 2021). For example, the benefits of incorporating digital technologies in the construction industry to support circular design concepts, improve material recycling rates, and reduce unnecessary construction waste have recently been highlighted by Talla and Stephen (2022).

In the fisheries and aquaculture sector, the use of Industry 4.0 technologies, such as IoT -enabled sensors and apps, to connect the net-to-plate supply chain, from farm or capture to customer's plate has the potential to improve planning, traceability, trackability, transparency, and control of factors related to weather conditions, illegal, unreported, and unregulated (IUU) fishing, and monitoring and management of seafood health, which can directly or indirectly lead to loss and waste (Garrett et al., 2019).

Application of Industry 4.0 technologies leads to the following waste advantages.

• Better seafood management through detailed data analysis.
• Real-time access to critical and accurate seafood-related information.
• Access to authentic seafood traceability data thereby improving consumer's confidence in the seafood products.

IUU fishing and overfishing are serious issues for seafood supply chain and Industry 4.0 technologies could provide details of catch including, species of animals caught, size and weight of catch. Besides, it informs when, where and on which boat the fish was caught (Garrett et al., 2019). Approximately half of the global population depends on protein obtained from seafood. Consequently, wild fish stocks are being rapidly depleted and aquaculture faces serious challenges (Natarajan and Ponnusamy 2022; Rowan 2022; Yue and Shen 2022). Furthermore, the waste and by-products generated from the seafood industry represents about 20% of total food processing waste (Sharma et al., 2022). AI has many real-world applications in support of zero-waste practices in seafood supply chains. AI may be used to learn from data (machine learning), identify patterns that may not be obvious to humans, and make decisions with little or no human intervention.

**Fig. 3:**
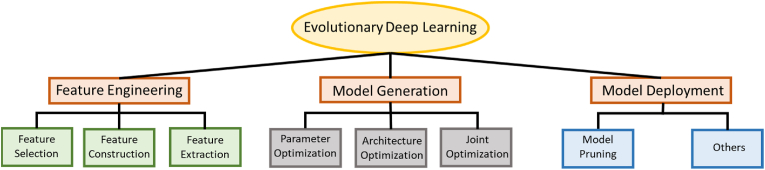
Evolutionary deep learning (Li et al., 2022).

The main machine learning tasks are classification, regression, and clustering (Al-Sahaf et al., 2019). Evolutionary machine learning has been the focus of significant interest in both academia and industry due to having a mechanism which automatically evolves towards optimal solutions unlike traditional deep learning which is heavily reliant on expert or domain knowledge that may not be readily available (Zhou et al., 2021). Evolutionary computation may be broadly divided into two categories: evolutionary algorithms and swarm intelligence (Al-Sahaf et al., 2019; Li et al., 2022). Deep learning is a subset of machine learning that uses artificial neural networks to mimic the learning process of the human brain. Fig. 3 describes the main components of evolutionary deep learning approaches.

Evolutionary machine learning techniques have been applied in various industries to reduce time and cost for both production and transportation (Tabrizi et al. 2018). Specifically, AI may enable better anticipation of supply for waste materials, and lessen unnecessary warehousing and potential shortages, which reduces costs and increases revenues, while helping to achieve a circular economy (Tseng et al., 2022).

Another Industry 4.0 technology that has shown great potential in improving the performance of food supply chains is IoT. This technology is a network of devices connected through sensors that can be located, identified, and operated upon (Ng and SusanWakenshaw, 2017). IoT allows better tracking and monitoring of food products and other resources, with important benefits in terms of operational and resource efficiency as well as traceability (Jagtap et al., 2021a, Jagtap et al., 2021b; Jagtap et al., 2021a, Jagtap et al., 2021b). IoT has also shown potential to support in reducing (Liegeard and Manning 2020) and valorizing food waste (Anbarasu et al. 2020).

Beside AI and IoT, other Industry 4.0 enabling technologies, such as BD (Belaud et al., 2019; Rejeb et al. 2022; Rejeb et al. 2021), have been shown to provide significant opportunities to reduce and valorize food waste and by-products. The opportunities provided by IoT used along with smart sensors to reduce food waste and by-products in the food supply chain have been recognized (Kuswandi et al. 2022; Zhu et al., 2022). More details about the application of these Industry 4.0 technologies in reduction and valorization of seafood waste and by-products are provided in the following sections.

In brief, digital transformation powered by Industry 4.0 technologies in seafood supply chain helps to increase connectivity, visibility, transparency, predictive capability, adaptability, and self-learning. This leads to a decreased seafood waste and optimal valorization pathways.

## 5 Applications of industry 4.0 in reduction and valorization of seafood waste

### 5.1 Artificial Intelligence (AI)

AI can be used for both reduction and valorization of seafood waste and by-products, as discussed in the following sections.

#### 5.1.1 Reduction of seafood waste using AI

AI-based approaches used to reduce seafood waste may be classified according to supply chain relation: harvest, processing, or consumption. Predicting food integrity issues using AI not only can prevent unsafe products from entering the food supply, but early detection facilitates the reduction of waste. Reducing waste across supply chains may be accomplished using fresh seafood forecasting models to align supply and demand based on machine learning models (Miguéis et al., 2022).

Aquaculture contributes significantly to satisfying the global demand for high quality seafood. Aquaculture enables the collection of large amounts of data that would be difficult to collect for wild stocks. Consequently, AI finds applications in different domains of aquaculture including environmental analysis, feed management, disease management, and fish stock assessment (Gladju and Kanagaraj 2021). Harmful algal blooms may produce toxins that accumulate in shellfish causing contamination. Machine learning forecasting tools are being developed to help the shellfish industry limit damages, improve mitigation measures, and reduce production losses (Cruz et al., 2021). A climate-driven predictive model using the elastic net machine learning method has been developed for the level of Vibrio parahaemolyticus (foodborne pathogen) in oysters from Taiwanese farms to help quantify the infection risk from eating this seafood (Ndraha and Hsiao 2022).

Pollutant contamination is another serious issue that can affect consumer health and cause seafood waste. Near-infrared spectroscopy combined with constrained difference extreme learning has been used to rapidly and accurately detect heavy metal contamination in mussels (Y. Liu et al., 2022a, Liu et al., 2022b). Microplastic adulteration detection in fish and seafood has been accomplished using attenuated total reflection mid-infrared spectroscopy in combination with machine learning techniques (Owen et al., 2021).

Seafood processing results in solid waste and wastewater generation which may cause major environmental risks (Singh et al., 2022). An anaerobic system for treating seafood processing wastewater has been modelled using multi-layer feed forward neural network capable of predicting chemical oxygen demand removal (Rashidi and Moghaddam 2021). The production of wastewater is also associated with higher energy consumption. AI may be effectively used for the prediction and optimization of energy use in seafood processing plants (Murali et al., 2021). Seafood processing such as crab processing is highly dependent on manual labor. Convolutional neural networks have been proposed to advance automated crab processing (Wang et al., 2018).

Food spoilage is a common cause of food waste. Extreme gradient boosting tree, a machine learning algorithm, has been used to predict the behavior of the pathogen Listeria monocytogenes present in seafood (Hiura et al. 2021). Probabilistic topic modelling has been used for volatilome-based seafood spoilage characterization of Atlantic salmon (Kuuliala et al., 2021). The spoilage of salmon and tuna has also been evaluated using a portable visible/near visible spectrometer using convolutional neural network-based machine learning (Moon et al., 2020). A paper chromogenic array coupled with a machine learning neural network has been used to detect viable pathogens in seafood via volatile organic compounds sensing (Yang et al., 2022). Deep convolutional neural networks have been applied to monitor shrimp freshness by recognizing their scent fingerprint (Ma et al., 2021).

#### 5.1.2 Valorization of seafood waste using AI

**Fig. 4:**
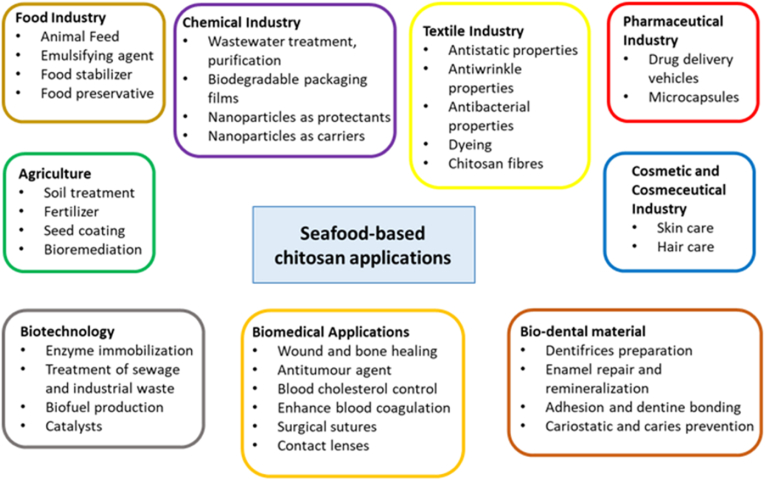
Applications of seafood-based chitosan in various industries (Tan et al., 2021).

Seafood waste management aids the long-term conservation of natural resources. Seafood waste and by-products also contain useful biomaterials, which have yet to be fully utilized due to inadequate waste disposal and solid waste management. Crustacean shells typically contain 15%–40% chitin, which is closely related to ingredients like protein, calcium carbonate, and lipids. Chitin and its main derivative, chitosan, are promising biomaterials, that are natural amino polysaccharide polymers. Chitin can be extracted either chemically or biologically for use in various industries as shown in Fig. 4 (Santos et al., 2020; Tan et al., 2021). Particle swarm optimization and artificial neural networks have been used for optimization of the fermentation medium for chitinase production (Suryawanshi and Satya Eswari, 2022).

Seafood waste and by-products are also a generally overlooked potential source of nano-carbons. Different kinds of nano-carbons such as graphene, carbon dots, carbon quantum dots, carbon nano onions, graphene quantum dots, carbon nanotubes, porous carbon, carbon nanomesh, and carbon aerogels can be prepared from seafood waste depending upon the synthesis environments and composition of precursors (Sharma et al., 2021). Green synthesized nano-carbons are a cost-effective alternative to traditionally manufactured nanoparticles. Computational nano-design using machine learning can be a low-cost means of screening and investigating a wide range of structural designs, including the stability of nanomaterials and their property prediction (Mekki-Berrada et al., 2021). AI has the potential to speed up biodot design and synthesis processes by e.g., searching and analyzing current databases to guide the selection of suitable precursors for different application needs such as biomedical imaging (Choi et al. 2020).

Valorizing available and high-potential by-products and side streams for the manufacture of value-added products supports the objectives of a circular economy. In this respect, seafood waste-derived renewable and biodegradable polymers have potential use in sustainable food packaging materials (Zhao, Li, and Du 2022). Specifically, edible films produced from fish gelatin and chitosan extracted from seafood waste have desirable antimicrobial and antioxidant properties for extending shelf-life and they are non-toxic (Debeaufort 2021). AI has been used for the classification and selection of fish gelatin packaging film (da Silva e Silva et al., 2021).

The microalgae industry currently utilizes expensive and energy-intensive harvesting technologies. Microalgae have potential as biofuel and alternative food sources. Fish bone waste has been identified as a novel bioflocculant for rapid microalgae harvesting in which an artificial neural network was employed to predict the complex processes involved (Suparmaniam et al., 2022). Details on the biology of microalgae consist of immense data so combining AI with mathematical modelling is a promising future direction for identifying key pathways for growth, protein production, and the improvement of taste and sensory properties (Helmy et al., 2022).

### 5.2 Big Data

Big data (BD) refers to large, complex, and changing datasets that cannot be processed with traditional methods (Ayed et al., 2022). Big data analytics (BDA) is the process of using advanced analytics on these datasets (Ciccullo et al., 2022). In the seafood sector, BD/BDA can be used to identify trends and patterns in the large amount of data produced (Jagtap and Duong 2019) resulting in improved efficiency, transparency, customer satisfaction and sustainability. This can directly or indirectly contribute to the reduction and valorization of waste and by-products, as discussed in the following sections.

#### 5.2.1 Optimization of production

BDA, together with the sensors and underwater image processing system, can be used to identify the exact feeding timing, dietary strategy, feed quantity and disease diagnosis needed to optimize production (Akerkar and Hong 2021). This will prevent waste in the early stages of aquaculture growth and improve the quality of seafood products. Along these lines it was discussed how marine salmon farming can be improved through the application of remote wireless data networks and digital dashboards, using BDA to interpret data obtained from various monitoring technologies (Bell et al., 2022). This can help meet the expectations of environmental responsibility, social responsibility, food safety and animal welfare in the supply chain. In addition, a solution was described using data from machine vision technology to improve the accuracy and efficiency of seafood sorting in production lines, specifically for large yellow croaker (Wu et al. 2019). Otherwise, data mining using BD-based data visualization was applied for sensory evaluation of Chinese mitten crabs, which is necessary for grading and can help target different market segments and avoid waste (Yang et al., 2021).

#### 5.2.2 Supply chain management

BD can be used to track and analyze the movement of seafood products throughout the supply chain, from the point of capture to the point of sale, using data from sensors and other tracking technologies. This can improve the efficiency and traceability of the supply chain, as well as reduce the risk of fraud, food safety incidents and waste. Thus, Al-Sahaf et al. (2019) discussed the application of evolutionary machine learning (EML) and BDA in various industries, including seafood transportation scheduling. These techniques can reveal high spoilage rates due to improper storage or transportation, which can lead to more waste. Besides, Riani et al. (2018) proposed a suitable method for use on large datasets and BDA to detect potentially fraudulent activities in seafood import data in the European Union, which can improve supply chain efficiency and reduce waste.

#### 5.2.3 Market analysis and customer strategies

BD/BDA can be used to monitor and manage household inventory and consumables, and inform retailers about consumption behavior, helping them develop strategies to plan inventory and reduce seafood wastage (Kuzmina et al., 2019). Websites and apps can be used to connect consumers and retailers with charities and food banks to improve the redistribution of seafood that would otherwise go to waste (Michelini et al. 2018). These by-products, such as heads, shells, and bones, often have valuable chemical and functional properties that can be exploited in other applications, such as in the production of feed, fertilizers, or nutraceuticals. BD has also been used to identify the factors contributing to the spatial imbalance of seafood restaurants in mainland China, which can help optimize their location and reduce potential seafood waste (Tian et al., 2021).

#### 5.2.4 Prospects for using BD to reduce and valorize seafood waste and by-products

The future use of BD in the seafood industry is expected to be more advanced and efficient in the reduction and valorization of waste and by-products (Table 1).

**Table 1:** Potential applications of BD/BDA to reduce and valorize seafood waste and by-products.

Application	Description	Example
Predictive analytics	Analyzing large datasets on factors such as production, supply chain and market trends to anticipate and avoid waste and by-product generation	Using data to predict when overproduction of a certain type of shellfish is likely to occur and take measures to avoid it
Real-time control	Using sensors and other tracking technologies to monitor operations in real time and identify and address waste and by-product generation as it occurs	Using sensors to monitor the freshness of seafood products and prevent spoilage
Collaborative platforms	Using BD to connect seafood companies with researchers, suppliers, and other stakeholders to share data and knowledge on waste and by-product reduction	Developing new technologies and best practices to reduce and valorize waste and by-products and extend these solutions to the whole industry

In summary, it is worth noting that although BD/BDA are not widespread in the seafood industry today, they have a great potential to reduce waste and by-product generation significantly. By harnessing the power of data analytics, seafood companies could become more efficient and sustainable, while creating new opportunities for growth and innovation.

### 5.3 The Internet of Things (IoT)

The main application of IoT in the fish and shellfish sector is in monitoring and automated adjustment of various aquaculture processes. In general, optimizing the aquaculture process allows improving the efficiency of the process, therefore reducing waste generation. An important advantage that IoT offers to aquaculture is the monitoring of water quality parameters. The parameters most commonly monitored via IoT are temperature, dissolved oxygen and pH (Prapti et al., 2022). Most of the IoT applications have been implemented for inland aquaculture, as opposed to marine aquaculture (Prapti et al., 2022).

A small-scale example of an IoT-supported aquaculture system was developed by Saha et al. (2018) by using Raspberry Pi, Arduino, various sensors, a camera and an Android application. Daud et al. (2022) used an IoT-based prototype to monitor the production of shrimps in aquaculture. Other small-scale systems have been developed to automatically release fish feed by an IoT-connected feeder (Akila et al., 2018; Janpla et al. 2019). Idachaba et al. (2017) combined both systems to automatically manage the automatic fish feeding system as well as the water quality monitoring of the pond with IoT. Some of these solutions have already been tested in fisheries. For instance, Huan et al. (2020) installed a water quality monitoring system based on narrow band IoT (NB-IoT) in a fishery in China and successfully measured temperature, pH and dissolved oxygen. A review on the recent developments of IoT-based aquaculture practices was prepared by Yadav et al. (2022).

**Fig. 5:**
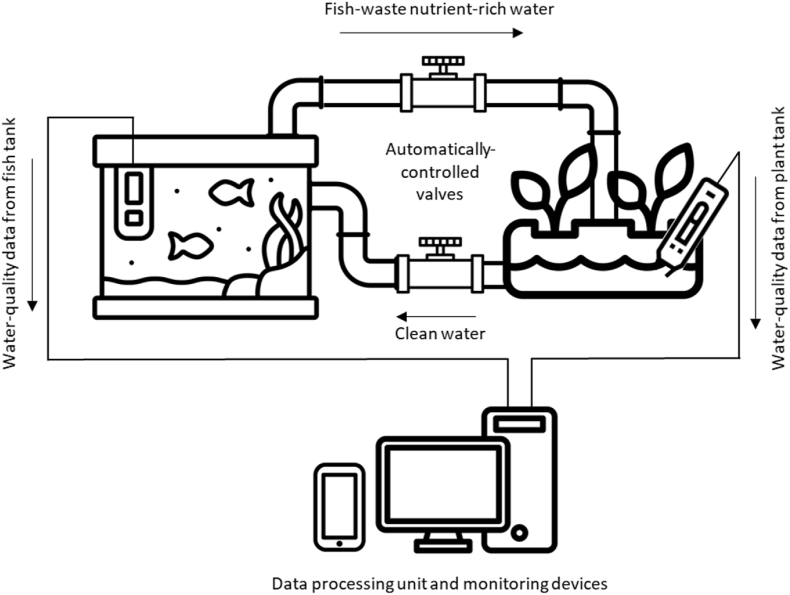
Aquaponics supported by IoT.

IoT has also started to be used to monitor and control aquaponics, i.e., the combination of aquaculture with hydroponics. In this way, the fish waste, that lowers the quality of the water where the fish grows, is removed, and used to provide essential nutrients to the plants. The water from the plants, once the nutrients have been absorbed by the plants, is returned to the fish tank. A schematic representation of this system is provided in Fig. 5. There are several successful examples of the use of IoT to support this process and recycle fish waste, e.g. (Aishwarya et al., 2018; Boonrawd et al. 2020; Dawa et al. 2022; Hari Kumar et al., 2016).

IoT-based systems have also been applied in the fish and shellfish sector in other areas, for instance to reduce energy and water consumption (Murali et al., 2021), monitor weight and location of fish at docks (Muslihi and Dani Achmad, 2019) and facilitate more accurate traceability (Gao et al. 2019; Kochanska 2020). An area with great potential to reduce waste in the sector is improved preservation methods, which increase the product shelf life and therefore reduce waste generation. Several preservation techniques can be monitored and controlled by IoT, including traditional techniques such as conventional freezing, drying, smoking, fermenting and salting; as well as newer techniques such as novel freezing methods (e.g. pressure, ultrasound, electrical and magnetic freezing), use of edible films and coatings, application of natural preservatives, and the use of nanotechnology (Hassoun et al., 2022a, Hassoun et al., 2022b, Hassoun et al., 2022c, Hassoun et al., 2022d, Hassoun et al., 2022e, Hassoun et al., 2022f, Hassoun et al., 2022g). Moreover, several parts of the discarded fish can be upcycled to obtain value from them. For instance, fish scale, a common waste material in fish markets, have already been used to produce electricity by triboelectric nanogenerators for self-powered sensors and IoT devices (Singh et al., 2023). A number of valuable compounds, such as enzymes, biomacromolecules and edible proteins can be extracted from fish waste and by-products and used to produce novel food ingredients, nutraceuticals, pharmaceuticals, biomedical materials, fine chemicals, fuels and other value-added products (Hassoun et al., 2021; Karkal and Kudre 2020, 2022; Nag et al., 2022; Shahidi et al., 2019). IoT can support the process of extracting such compounds and processing them into new materials by monitoring and acting upon the process, as well as collecting and sharing live information between the different stakeholders in the supply chain.

### 5.4 Smart sensors

Smart sensors combine data acquisition, internal processing, and transmission for decision-making. These sensors are generally robust, flexible, and miniaturized to be adapted to different food products, environments, and conditions (e.g., abrasive, dusty, noisy, cold, hot, and wet). Nowadays sensors benefit from Industry 4.0 technologies, allowing them to match these different requirements and increase their potential of data acquisition, storage, and processing. The combination of sensors or a sensors network with multivariate data analysis methods, machine learning, and IoT can improve selectivity, food process monitoring and traceability (tracking) in real time from farm to fork. This can contribute to better quality, productivity, ethics, reduction and management of seafood waste and by-products and finally better global sustainability. Some examples of smart sensors are presented in Table 2. Many recent publications have highlighted the importance of different types of sensors for various applications in food quality and safety monitoring (Lin et al., 2023; Meira et al., 2023; Selva Sharma et al., 2023).

**Table 2:** Use of different smart sensors for fish quality evaluation.

Smart Sensors	Objective	Technique used	Sample tested	Authors
CUPRAC-Nafion	Freshness monitoring	Colorimetry	Sea bass (*Dicentrarchus labrax*)	(Avan et al., 2023)
Multicolor biosensor based on the etching of gold nanorods (GNRs)	Freshness monitoring	Colorimetry	Miichthys minuend Whitebait	(Chen et al., 2017)
Hydrogel coating flexible pH sensor with a flexible battery-free wireless electronic system	Freshness monitoring	pH-sensitive electrode	Fish	(Mu et al., 2022)
Hydrogel-pH-electrode based wireless sensor	Freshness monitoring	pH-sensitive electrode	Tilapia fish fillets	(Bhadra et al., 2015)
Alginate beads of “Laminaria digitata” brown alga	Freshness monitoring	Colorimetry	Rainbow trout	(Majdinasab et al., 2018)
Hydrogel composed of a colorimetric substance (Au@MnO2) and reducing agent precursor (β-D-glucose pentaacetate	Freshness monitoring	Smartphone RGB analysis-based colorimetric sensor	Grass carp, crucian carp, and grouper	(Zhang et al., 2021)
Nano plasmonic membrane	Freshness monitoring	UV–Vis absorbance	Fish	(Heli et al., 2016)
Ultrasensitive Porous-Electrode-Capped Organic Gas Sensor System	Freshness monitoring	Resistive gas sensor	Mackerel	(Chang et al., 2017)
E-Nose	Freshness monitoring	Dioxide gas sensors	Rainbow trout	(Vajdi et al., 2019)
E-Nose	Fish authentication	Eight metal oxide sensors	Anchovy, Horse Mackerel, Whiting	(Güney and Atasoy 2015)
Portable E-nose	Detect and classify a fish meal of different qualities	Ten MOS (metal oxide semiconductor) gas sensor	6 fish meal samples	(Li et al., 2019)
E-tongue	Freshness monitoring	Seven sensors array	Crucian carp	(Han et al., 2015)
E-tongue + E-nose	Discrimination of flavors and tastes of traditional Chinese fish soups	E-nose: commercial FOX4000 sensor array system	Silver carp fish soups	
		E-tongue: α-ASTREE II Liquid Taste Analyzer		
Hyperspectral imaging	Detection of blood in fish muscles	Vis-NIR-640 imaging spectrograph 430–1000 nm	Cod fillets	(Skjelvareid et al., 2017)
Hyperspectral imaging	Prediction of textural changes	Vis-NIR push broom spectrograph 400–1000 nm	Grass carp	(Ma et al., 2017)
Hyperspectral imaging	Prediction of microplastics contamination	HyperSpec NIR system 900–1700 nm	Crucian carps	(Zhang et al., 2019)

#### 5.4.1 Spectrochemical sensors

**Fig. 6:**
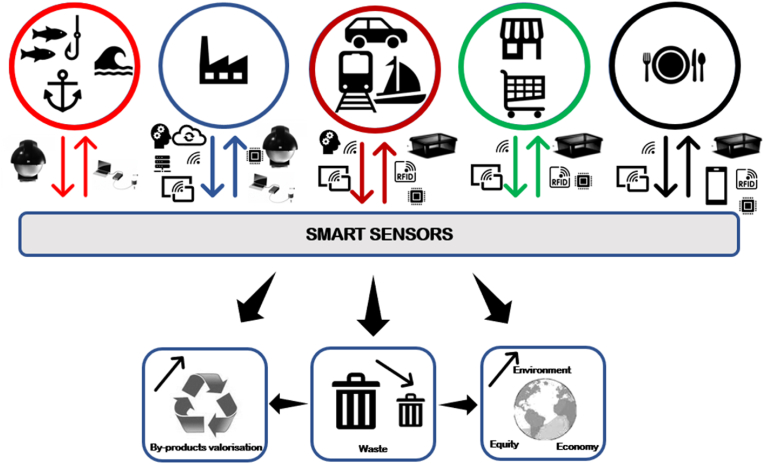
Use of smart sensors in reduction and valorization of seafood waste and by-products.

Smart spectroscopic sensors are promising candidates for reducing seafood waste and by-products (Fig. 6). These molecular fingerprinting technologies are sensitive to chemical interaction and vibrations of food constituents (e.g., proteins, fat, polysaccharides, sugar, etc.) (Hassoun, Abdullah, Aït-kaddour, Ayşegül Beşir, et al. 2022). Near-Infrared (NIR), Mid-Infrared (MIR), fluorescence, Raman, multispectral (MSI) and hyperspectral imaging (HSI) are among those techniques. These techniques can be miniaturized and designed to support industrial environments conditions (e.g., waterproof, surviving drops, and temperature fluctuation).

##### 5.4.1.1 Spectrochemical sensors for seafood waste reduction

A major objective to improve the overall value of food, reduce food waste or the generation of by-products is the premature detection of spoilage. Spectrochemical techniques have been used in different studies to tackle this issue. For example, the spoilage of salmon and tuna has been evaluated by using a portable smart visible/near handheld spectrometer coupled with convolutional neural network based on machine learning algorithm (Moon et al., 2020). The device is already commercially available (LinkSquare®) and can be used directly by consumers to know whether the food is safe and still appropriate for consumption, avoiding economic and environmental impact.

Electrochemical impedance spectroscopy (EIS) and terahertz time domain spectroscopy (TDS) have also demonstrated their interest to monitor spoilage (Zhang et al., 2022). The two techniques permitted to distinguish four stages of salmon deterioration. However, THz TDS in reflection mode was the most promising for industrial applications due to their non-invasive and non-destructive potential. In addition, some studies reported the interest of HSI to assess the presence and growth of microorganisms (Cheng and Sun, 2015a, 2015b) in fish. Zhang et al. (2019) proposed a promising (recall and precision factors >96.22%) in-line HSI technique for future industrial applications to quantify and identify microplastics (PE, PS, PET, PP, and PC) in fish. In another study NIR spectroscopy combined with constrained difference extreme learning has been used to rapidly and accurately detect heavy metal contamination in mussels (Y. Liu et al., 2022a, Liu et al., 2022b).

##### 5.4.1.2 Spectrochemical sensors for seafood waste management

Resource depletion and global warming call for more efficient recycling of biogenic material waste resulting from aquaculture and seafood industry operations. In this context, Ogresta et al. (2021) proposed a simple MIR absorbance ratio ((υ(CH_2,3_) band and the υ_asym_ (CO_3_^2−^)) to differentiate, based on their composition, various biogenic powders from raw or food-processed shellfish shells. The proposed method can be useful for decision making regarding the development of advanced applications of biogenic powders (e.g., pharmaceutical carriers, powders loaded with various fluids for new composite materials, or adsorbents for environmental decontamination). In another study, Nieto-Ortega et al. (2022) proposed a handheld NIR sensor combined with multivariate data analysis (PLS-R) to predict the content and composition of fish oil by-products. Good fitting models (0.97 ≤ R^2^ ≤ 0.99) in real conditions were obtained for the prediction of SFAs, MUFAs, PUFAs and ω-3 fatty acids. This device was proposed to reduce the volume of discarded fish by-products that are obtained directly after fishing. Due to their high nutritive value, it is possible to give them an extended life by correctly orienting them towards the best recycling process.

#### 5.4.2 Electrochemical sensors for seafood waste reduction

Electrochemical sensors have the capability of transforming information perceived by a receptor into an electric signal thanks to a transducer element. Based on the type of signal transduction, electrochemical sensors can be referred to as voltametric (or amperometric), potentiometric, impedimetric, and conductometric (Oveissi et al., 2022). Electronic Nose (E-Nose) and Tongue (E-Tongue) are electrochemical sensors devices that mimic the functions of human nose and taste. These smart sensors can contribute to potential reduction of fish waste and fish by-products by digitalizing human sense and providing rapid, accurate, and reproducible measurements. E-Tongue and E-Nose have been widely used in the evaluation of fish freshness, with different applications and results based on the characteristic of the sensors. For example, Grassi et al. (2019) developed a low-cost and portable E-Nose, named Mastersense, combined with K-Nearest Neighbours’ algorithm to assess fish freshness. The Mastersense device gave a good predictive model for the classification of salmon samples based on their freshness (sensitivity and specificity >0.75). In a recent study, Li et al. (2023) proposed an original approach based on data fusion technology and four machine learning algorithms (ANN, RFR, XGBoost, and SVR) to combine the E-nose, E-tongue, and E-eye (colorimeter) data signals in order to predict freshness of horse mackerel during frozen storage (90 days). All the predictive models exhibited R^2^ values of prediction ≥0.835.

The growing interest in IoT, smart homes, wearable devices, and point-of-need sensors has been the main driver fueling the development of new classes of sensors such as printed electrical gas sensors. Recently, Barandun et al. (2019) proposed a wireless on/off paper-based electrical gas sensor (cellulose fibers) connected to an RFID tag to sense ammonia and carbon dioxide gases release during fish spoilage. This near zero-cost technology (battery-less) can be integrated into food packaging to monitor freshness and reduce food waste or implemented into near-field-communication tags that can be interrogated with smartphones.

#### 5.4.3 Colorimetric sensors for seafood waste reduction

Technology based on colorimetric sensors can be integrated into packaging and help monitoring food freshness or other quality parameters due to color change of the product. They offer the simplest and most practical way to monitor freshness. Those sensors can be used to detect gases (Crowley et al., 2005), volatile organic compounds (Lv et al., 2019; Magnaghi et al., 2020), toxic molecules (Xu et al., 2022), heavy metals (Swain et al. 2020), and biomolecules (Calabretta et al., 2023), which can provide a real-time freshness quality index of the packaged fish and seafood. Many examples in recent literature follow this idea. Colorimetric sensors can be very useful for food end-users and help avoid wasting safe food products or consumption of unsafe ones. For example, Valdez et al. (2019) and Teymouri and Shekarchizadeh (2022) proposed smart sensors based solely on visual observation of color change depending on fish freshness. In the study by Teymouri and Shekarchizadeh (2022), fish freshness was evaluated by a copper nanoparticle sensitive to volatile sulfur compounds. However, the evaluation of color shift by naked eyes can be subjective and at the best semiquantitative. To tackle this issues, Zhang et al. (2021) proposed to match smartphone and colorimetric sensor technologies for real time monitoring of fish freshness during storage. The authors developed a gold-derived inorganic nanocomposites-based (Au@MnO2 nanocomposites) sensing hydrogel which color shifts in the presence of TVB-N. The changes in color intensity can be evaluated by a smartphone combined with RGB analysis.

## 6 Conclusion and future perspectives

Seafood waste is becoming an urgent environmental, social, and economic challenge that needs to be overcome. The latest FAO report on fisheries and aquaculture reveals that the world production of fishery and aquaculture products reached 214 million tons in 2020, including 178 million tons of aquatic animals (Zhao et al., 2022). Unfortunately, much of the production is discarded due to high perishability, small size, unappealing shape and lack of adequate infrastructure, particularly on-board fishing vessels. It is estimated that the share of fishery discards amounts to about 10 million tons, whereas industrial seafood waste amounts to 15 million tons and it is increasing. The growth in seafood waste and by-products appears to be linked to the increased demand for ready-to-eat seafood products, in which up to 70% of the total wet weight is discarded, especially in the case of crabs, shrimps, and lobsters. Therefore, the problem is primarily linked to environmental challenges, but ethical and social issues are still also unresolved despite good intentions.

This review discusses the latest successful applications of Industry 4.0 technologies that can be used to reduce and valorize seafood waste and by-products, and secure and foster resilience in fishery ecosystems while promoting a circular and bold reorganization of the fish supply chain. This could enhance seafood sustainability and provide opportunities in line with the blue transformation vision, helping to meet several UN's Sustainable Development Goals by 2030. The potential of Industry 4.0 technologies and digital innovations (such as BD, AI, IoT and smart sensors) to reduce food waste has been demonstrated. For example, Industry 4.0 technologies can help to replace traditional “best before” dates on food products with advanced, low-cost optical/colorimetric sensors that are objectively able to distinguish unspoiled seafood products from those that are spoiled. It is estimated by the World Economic Forum estimates that blockchain, AI, sensors and IoT could save up to 85 millions of tons of food waste by 2030 (Billant 2021; Nayyar et al., 2018). IoT-enabled sensors and apps for product traceability may thus also resolve the long-standing issue of IUU fishing, with very interesting implications in terms of reducing discards in fisheries. Within the context of industrial seafood processing, AI and machine learning are providing important tools and applications to make decisions without human intervention and reduce food waste. Moreover, AI- and BD-based approaches have been used for monitoring and treating seafood processing wastewater and have proved essential in varied aquaculture sectors, especially in environmental analysis and feed and disease management, helping improve mitigation measures and reduce production losses. As for food safety, near-infrared spectroscopy and machine learning techniques are proving to be essential in rapidly and accurately detecting heavy metals and microplastics in many seafood products. Machine learning algorithms and probabilistic topic modelling could also be applied to predict microbiological and (bio)chemical spoilage, respectively. An increased adoption of Industry 4.0 technologies and digital tools is necessary to achieve more sustainable food waste management.

Considering the potential of seafood waste and fishery discards as sources of valuable nutrients, these should be used to produce high value-added food, e.g., “Ready-to-Use Therapeutic Food”. By-products from initial processing could then be used to produce numerous functionally active compounds (e.g., chitin, carotenoids, collagen, etc.) and new biomaterials (such as carbon nanofibers); in the end, residual materials could be used to produce biogas and compost, in keeping with the model of circular economy and green technology.

Despite the high potential of Industry 4.0 technologies and digital tools, their application in the seafood industry and other food sectors is still in the innovation phase and facing several challenges. One of the main obstacles is lack of connectivity and adequate digital infrastructure, especially in the case of smallholder farmers and fishermen engaged along the seafood supply chain. Therefore, there is a need to develop digital applications and create suitable digital platforms to support aquaculture farmers and other value chain actors. Another challenge is that digitalization and adoption of new technologies can often be perceived as costly and risky in various agriculture and food industries, including the fisheries and aquaculture sector. Other issues related to data security and privacy, lack of skilled labors, in addition to policy and legal constraints have been identified in many of the reviewed studies and should be addressed to promote a wider use of Industry 4.0 technologies and digital tools along the seafood supply chain.

Many of the discussed challenges could be solved through strong partnerships and close collaboration between the different actors in supply and value chains of fisheries and aquaculture (fish farmers, processors, distributors, consumers, etc.). Adequate funding for training and upskilling initiatives and raising consumer awareness of food waste is also needed. Moreover, providing technical and financial support for establishing digital infrastructure and logistics and robust digital policies are key elements to foster and accelerate digital transformation.

It is believed, in the lens of the ongoing, continuous, and accelerating progress that all Industry 4.0 technologies presented in this review could be applied more broadly to develop new food products and ingredients from fishery discards, providing these for countries with increasing populations that are battling child malnutrition, and reducing the carbon footprint of seafood waste. New digital innovations are expected to emerge in the near future and greater implementation of Industry 4.0 technologies is likely to be achieved in the seafood sector, accelerating the move toward the fifth industrial revolution.

## Funding

This research received no external funding.

## CRediT authorship contribution statement

**Abdo Hassoun:** Conceptualization, Methodology, Study design, Resources, Data curation, Writing – original draft, Writing – review & editing, Supervision, Project administration. **Janna Cropotova:** Conceptualization, Methodology, Study design, Resources, Data curation, Writing – original draft, Writing – review & editing, Supervision, Project administration, Funding acquisition. **Hana Trollman:** Methodology, Study design, Resources, Data curation, Writing – original draft, Writing – review & editing. **Sandeep Jagtap:** Methodology, Study design, Resources, Data curation, Writing – original draft, Writing – review & editing. **Guillermo Garcia-Garcia:** Methodology, Study design, Resources, Data curation, Writing – original draft, Writing – review & editing. **Carlos Parra-López:** Methodology, Study design, Resources, Data curation, Writing – original draft, Writing – review & editing. **Nilesh Nirmal:** Methodology, Study design, Resources, Data curation, Writing – original draft, Writing – review & editing. **Fatih Özogul:** Methodology, Study design, Resources, Data curation, Writing – original draft, Writing – review & editing. **Zuhaib Bhat:** Methodology, Study design, Resources, Data curation, Writing – original draft, Writing – review & editing. **Abderrahmane Aït-Kaddour:** Methodology, Study design, Resources, Data curation, Writing – original draft, Writing – review & editing. **Gioacchino Bono:** Methodology, Study design, Resources, Data curation, Writing – original draft, Writing – review & editing.

## Declaration of competing interest

The authors declare the following financial interests/personal relationships which may be considered as potential competing interests: Janna Cropotova reports article publishing charges was provided by Norwegian University of Science and Technology.

## Data Availability

No data was used for the research described in the article.

## References

[bib1] Abbate Stefano, Centobelli Piera, Cerchione Roberto (2023). The digital and sustainable transition of the agri-food sector. Technol. Forecast. Soc. Change.

[bib2] Abuine Racheal, Rathnayake Anuruddhika Udayangani, Byun Hee Guk (2019). Biological activity of peptides purified from fish skin hydrolysates. Fish. Aquat. Sci..

[bib3] Agustiono Tonni, Meidiana Christia, Hafiz Mohd, Othman Dzarfan, Hwang Hui, Wayne Kit (2023). Strengthening waste recycling industry in malang (Indonesia): lessons from waste management in the era of industry 4.0. J. Clean. Prod..

[bib4] Aishwarya K.S., Harish M., Prathibhashree S., Panimozhi K. (2018). Proceedings of the International Conference on Inventive Communication and Computational Technologies.

[bib5] Akerkar R., Hong M. (2021). Big data in aquaculture: opportunities and challenges for sogn og fjordane region. Vestlandsforsking.

[bib6] Akila I.S., Karthikeyan P., Hari Haran M.V., Hari Krishnan J. (2018). Proceedings of the 2nd International Conference on Electronics, Communication and Aerospace Technology.

[bib7] Al-Sahaf Harith, Bi Ying, Chen Qi, Lensen Andrew, Mei Yi, Sun Yanan, Tran Binh, Xue Bing, Zhang Mengjie (2019). A survey on evolutionary machine learning. J. Roy. Soc. N. Z..

[bib8] Ali Ahtisham, Wei Shuai, Liu Zhenyang, Fan Xiuping, Sun Qinxiu, Xia Qiuyu, Liu Shucheng, Hao Jiming, Deng Chujin (2021). Non-thermal processing technologies for the recovery of bioactive compounds from marine by-products. Lebensm. Wiss. Technol..

[bib9] Ananey-Obiri Daniel, Matthews Lovie G., Tahergorabi Reza, Galanakis Charis M. (2019). Proteins: Sustainable Source, Processing And Applications.

[bib10] Anbarasu V., Karthikeyan P., Anandaraj S.P. (2020). 2020 6th International Conference on Advanced Computing and Communication Systems.

[bib11] Annosi Maria Carmela, Brunetta Federica, Bimbo Francesco, Kostoula Marianthi (2021). Digitalization within food supply chains to prevent food waste. Drivers, barriers and collaboration practices. Ind. Market. Manag..

[bib13] Avan Aslı Neslihan, Karakaş Özge, Demirci-Çekiç Sema, Apak Reşat (2023). Enzymatic determination of hypoxanthine in fish samples as a freshness indicator using the CUPRAC colorimetric sensor. Enzym. Microb. Technol..

[bib14] Ayed, Ben Rayda, Hanana Mohsen, Ercisli Sezai, Karunakaran Rohini, Ahmed Rebai, Moreau Fabienne (2022). Integration of innovative technologies in the agri-food sector: the fundamentals and practical case of DNA-based traceability of olives from fruit to oil. Plants.

[bib15] Bader Farah, Rahimifard Shahin (2018). Proceedings ISCSIC’18.

[bib16] Barandun Giandrin, Soprani Matteo, Naficy Sina, Grell Max, Kasimatis Michael, Chiu Kwan Lun, Ponzoni Andrea, Güder Firat (2019). Cellulose fibers enable near-zero-cost electrical sensing of water-soluble gases. ACS Sens..

[bib17] Belaud Jean Pierre, Prioux Nancy, Vialle Claire, Sablayrolles Caroline (2019). Big data for agri-food 4.0: application to sustainability management for by-products supply chain. Comput. Ind..

[bib18] Bell Jennifer L., Mandel Randy, Brainard Andrew S., Altschuld Jon, Wenning Richard J. (2022). Environmental monitoring tools and strategies in salmon net-pen aquaculture. Integrated Environ. Assess. Manag..

[bib19] Benyam Addisalem (Addis), Tammara Soma, Fraser Evan (2021). Digital agricultural technologies for food loss and waste prevention and reduction: global trends, adoption opportunities and barriers. J. Clean. Prod..

[bib20] Bhadra Sharmistha, Narvaez Claudia, Thomson Douglas J., Bridges Greg E. (2015). Non-destructive detection of fish spoilage using a wireless basic volatile sensor. Talanta.

[bib21] Billant Jean (2021). Cutting edge technologies to end food waste. Food Sci. Technol..

[bib22] Boonrawd Patcharapol, Nuchitprasitchai Siranee, Nilsiam Yuenyong (2020). Aquaponics systems using Internet of Things. Adv. Intell. Syst. Comput..

[bib23] Boyd Claude E., McNevin Aaron A., Davis Robert P. (2022). The contribution of fisheries and aquaculture to the global protein supply. Food Secur..

[bib24] Calabretta Maria Maddalena, Gregucci Denise, Desiderio Riccardo, Michelini Elisa (2023). Colorimetric paper sensor for food spoilage based on biogenic amine monitoring. Biosensors.

[bib25] Cane Massimo, Parra Carmen (2020). Digital platforms: mapping the territory of new technologies to fight food waste. Br. Food J..

[bib26] Chang Liang-Yu, Chuang Ming-Yen, Zan Hsiao-Wen, Meng Hsin-Fei, Lu Chia-Jung, Yeh Ping-Hung, Chen Jian-Nan (2017). One-minute fish freshness evaluation by testing the volatile amine gas with an ultrasensitive porous-electrode-capped organic gas sensor system. ACS Sens..

[bib27] Chen Zhitao, Yue Lin, Ma Xiaoming, Guo Longhua, Qiu Bin, Chen Guonan, Lin Zhenyu (2017). Multicolor biosensor for fish freshness assessment with the naked eye. Sensor. Actuator. B Chem..

[bib28] Cheng Jun Hu, Sun Da Wen (2015). Rapid quantification analysis and visualization of Escherichia coli loads in grass carp fish flesh by hyperspectral imaging method. Food Bioprocess Technol..

[bib29] Cheng Jun Hu, Sun Da Wen (2015). Recent applications of spectroscopic and hyperspectral imaging techniques with chemometric analysis for rapid inspection of microbial spoilage in muscle foods. Compr. Rev. Food Sci. Food Saf..

[bib30] Choi Yoonah, Zheng Xin Ting, Tan Yen Nee (2020). Bioinspired carbon dots (biodots): emerging fluorophores with tailored multiple functionalities for biomedical, agricultural and environmental applications. Mol. Syst. Des. Eng..

[bib31] Ciccullo Federica, Fabbri Margherita, Abdelkafi Nizar, Pero Margherita (2022). Exploring the potential of business models for sustainability and Big data for food waste reduction. J. Clean. Prod..

[bib32] Coppola Daniela, Lauritano Chiara, Esposito Fortunato Palma, Riccio Gennaro, Rizzo Carmen, de Pascale Donatella (2021). Fish waste: from problem to valuable resource. Mar. Drugs.

[bib33] Crew Sterling (2021). Share more waste less. Food Sci. Technol..

[bib34] Crowley K., Pacquit A., Hayes J., Lau King Tong, Diamond D. (2005). IEEE Sensors.

[bib35] Cruz Rafaela C., Pedro Reis Costa Susana Vinga, Ludwig Krippahl, Lopes Marta B. (2021). A review of recent machine learning advances for forecasting harmful algal blooms and shellfish contamination. J. Mar. Sci. Eng..

[bib36] da Silva e Silva, Natácia Fabricio, Farias de Souza, Madson dos Santos Freitas Mauricio, Pino Hernández Enrique José Gregório, Dantas Vanderson Vasconcelos, Oliveira Marcos Enê Chaves, Sarkis Peixoto Joele Maria Regina, Henriques Lourenço Lúcia de Fátima (2021). Artificial intelligence application for classification and selection of fish gelatin packaging film produced with incorporation of palm oil and plant essential oils. Food Packag. Shelf Life.

[bib37] Daud Haji, Safwan Nasrun Mohammad, Kumar Patchmuthu Ravi, Au Thien Wan (2022). ACM International Conference Proceeding Series.

[bib38] David Love O., Nwulu Nnamdi I., Aigbavboa Clinton O., Adepoju Omoseni O. (2022). Integrating fourth industrial revolution (4IR) technologies into the water, energy & food nexus for sustainable security: a bibliometric analysis. J. Clean. Prod..

[bib39] Dawa Maritel, Lausa Samson M., Tibon Maria Rodelyn (2022). 3rd International Conference on Automation, Mechatronics, and Robotics.

[bib40] Debeaufort Frédéric (2021). Active biopackaging produced from by-products and waste from food and marine industries. FEBS Open Biol..

[bib41] Fan Weiwei, Tan Xiaoyi, Xu Xianbing, Li Guodong, Wang Zhenyu, Du Ming (2019). Relationship between enzyme, peptides, amino acids, ion composition, and bitterness of the hydrolysates of Alaska pollock frame. J. Food Biochem..

[bib42] FAO (2020).

[bib43] Gao Guandong, Xiao Ke, Chen Miaomiao (2019). An intelligent IoT-based control and traceability system to forecast and maintain water quality in freshwater fish farms. Comput. Electron. Agric..

[bib44] Gao Ruichang, Yu Qingqing, Shen Yang, Chu Qian, Chen Ge, Fen Siyu, Yang Mingxuan, Yuan Li, McClements David Julian, Sun Quancai (2021). Production, bioactive properties, and potential applications of fish protein hydrolysates: developments and challenges. Trends Food Sci. Technol..

[bib45] Garrett Angus, Cooper Lee, Lewis Tattersall (2019).

[bib46] Gladju J., Kanagaraj A. (2021). 2021 International Conference On Advancements In Electrical, Electronics, Communication, Computing And Automation.

[bib47] Grassi Benedetti, Opizzio Nardo, Buratti (2019). Meat and fish freshness assessment by a portable and simplified electronic nose system (Mastersense). Sensors.

[bib48] Gui Meng, Gao Liang, Rao Lei, Li Pinglan, Zhang Ying, Han Jia Wei, Li Jun (2022). Bioactive peptides identified from enzymatic hydrolysates of sturgeon skin. J. Sci. Food Agric..

[bib49] Gulzar Saqib, Benjakul Soottawat (2020). Impact of pulsed electric field pretreatment on yield and quality of lipid extracted from cephalothorax of pacific white shrimp (Litopenaeus vannamei) by ultrasound-assisted process. Int. J. Food Sci. Technol..

[bib50] Güney Selda, Atasoy Ayten (2015). Study of fish species discrimination via electronic nose. Comput. Electron. Agric..

[bib51] Han Fangkai, Huang Xingyi, Teye Ernest, Gu Haiyang (2015). Quantitative analysis of fish microbiological quality using electronic tongue coupled with nonlinear pattern recognition algorithms. J. Food Saf..

[bib52] Han Jia Wei, Zuo Min, Zhu Wen Ying, Zuo Jin Hua, En Li Lü, Yang Xin Ting (2021). A comprehensive review of cold chain logistics for fresh agricultural products: current status, challenges, and future trends. Trends Food Sci. Technol..

[bib53] Hari Kumar N., Baskaran Sandhya, Hariraj Sanjana, Krishnan Vaishali (2016). Proceedings - 2016 4th International Conference on Future Internet of Things and Cloud Workshops.

[bib54] Hassoun Abdo, Rustad Turid, Bekhit Alaa El-Din A. (2021).

[bib55] Hassoun Abdo, Alhaj Abdullah Nour, Aït-kaddour Abderrahmane, Beşir Ayşegül, Zannou Oscar, Önal Begüm, Aadil Rana Muhammad, Lorenzo Jose M., Khaneghah Amin Mousavi, Regenstein Joe M. (2022). Food traceability 4.0 as part of the fourth industrial revolution: key enabling technologies. Crit. Rev. Food Sci. Nutr..

[bib56] Hassoun Abdo, Aït-kaddour Abderrahmane, Adnan M., Abu-mahfouz, Rathod Nikheel Bhojraj, Bader Farah, Barba Francisco J., Cropotova Janna, Galanakis Charis M., Jambrak Anet Režek, Lorenzo M., Måge Ingrid, Ozogul Fatih, Joe Regenstein, Rathod Bhojraj, Bader Farah, Barba Francisco J., Biancolillo Alessandra, Cropotova Janna, Galanakis M., Jambrak Anet Režek, Lorenzo José M., Måge Ingrid, Ozogul Fatih, Joe Regenstein (2022). The fourth industrial revolution in the food industry — Part I: industry 4.0 technologies. Crit. Rev. Food Sci. Nutr..

[bib57] Hassoun Abdo, Bekhit Alaa El-din, Jambrak Anet Režek, Regenstein Joe M., Chemat Farid, Morton James D., Gudjónsdóttir María, Carpena María, Prieto A., Varela Paula, Arshad Rai Naveed, Aadil Rana Muhammad, Øydis Ueland Abdo Hassoun Alaa El-din Bekhit Anet Režek Jambrak, Regenstein Joe M., Chemat Farid, Morton James D., Gudjónsdóttir María, Carpena María, Prieto Miguel A., Arshad Rai Naveed, Aadil Rana Muhammad, Bhat Zuhaib, Ueland Øydis (2022). The fourth industrial revolution in the food industry — Part II : emerging food trends trends. Crit. Rev. Food Sci. Nutr..

[bib58] Hassoun Abdo, Cropotova Janna, Trif Monica, Rusu Alexandru Vasile, Bobi Otilia, Ahmad Nayik Gulzar, Jagdale Yash D., Saeed Farhan, Afzaal Muhammad, Joe M., Regenstein (2022). Consumer acceptance of new food trends resulting from the fourth industrial revolution technologies: a narrative review of literature and future perspectives. Front. Nutr..

[bib59] Hassoun Abdo, Prieto Miguel A., Carpena María, Bouzembrak Yamine, Hans J., Marvin P., Pallar Noelia, Barba Francisco J., Punia Sneh, Chaudhary Vandana, Ibrahim Salam, Bono Gioacchino (2022). Exploring the role of green and industry 4.0 technologies in achieving sustainable development Goals in food sectors. Food Res. Int..

[bib60] Hassoun Abdo, Anusha Siddiqui Shahida, Smaoui Slim, Ucak İ., Arshad Naveed, Bhat Zuhaib F., Bhat Hina F., Carpena María, Miguel A., Aït-kaddour Abderrahmane, Jorge A., Pereira M., Zacometti Carmela, Tata Alessandra, Ibrahim Salam A., Ozogul Fatih, Camara José S., Arshad Naveed, Bhat Zuhaib F., Bhat Hina F., Carpena María, Prieto Miguel A., Jorge A., Pereira M., Zacometti Carmela, Tata Alessandra, Ibrahim Salam A. (2022). Emerging technological advances in improving the safety of muscle foods: framing in the context of the food revolution 4.0. Food Res. Int..

[bib61] Hassoun Abdo, Anusha Siddiqui Shahida, Smaoui Slim, Ucak İlknur, Arshad Rai Naveed, Garcia-Oliveira Paula, Prieto Miguel A., Aït-Kaddour Abderrahmane, Rosa Perestrelo, Câmara José S., Bono Gioacchino (2022). Seafood processing, preservation, and analytical techniques in the age of industry 4.0. Appl. Sci..

[bib62] Hassoun Abdo, Jagtap Sandeep, Garcia-garcia Guillermo, Trollman Hana, Pateiro Mirian, Lorenzo M., Trif Monica, Vasile Alexandru, Muhammad Rana, Simat Vida, Cropotova Janna, C S. (2023). Food quality 4.0: from traditional approaches to digitalized automated analysis. J. Food Eng..

[bib63] Heli B., Morales-Narváez E., Golmohammadi H., Ajji A., Merkoçi A. (2016). Modulation of population density and size of silver nanoparticles embedded in bacterial cellulose via ammonia exposure: visual detection of volatile compounds in a piece of plasmonic nanopaper. Nanoscale.

[bib64] Helmy Mohamed, Elhalis Hosam, Liu Yan, Chow Yvonne, Kumar Selvarajoo (2022). Perspective: multi-omics and machine learning help unleash the alternative food potential of microalgae. Adv. Nutr..

[bib65] Hemker, Kumar Ashutosh, Nguyen Loc Thai, Karwe Mukund, Salvi Deepti (2020). Effects of pressure-assisted enzymatic hydrolysis on functional and bioactive properties of Tilapia (Oreochromis niloticus) by-product protein hydrolysates. Lebensm. Wiss. Technol..

[bib66] Hiura Satoko, Koseki Shige, Koyama Kento (2021). Prediction of population behavior of Listeria monocytogenes in food using machine learning and a microbial growth and survival database. Sci. Rep..

[bib67] Hjellnes Veronica, Rustad Turid, Falch Eva (2020). The value chain of the white fish industry in Norway: history, current status and possibilities for improvement – a review. Reg. Stud. Mar. Sci..

[bib68] Hou Yongqing, Wu Zhenlong, Dai Zhaolai, Wang Genhu, Wu Guoyao (2017). Protein hydrolysates in animal nutrition: industrial production, bioactive peptides, and functional significance. J. Anim. Sci. Biotechnol..

[bib69] Huan Juan, Li Hui, Wu Fan, Cao Weijian (2020). Design of water quality monitoring system for aquaculture ponds based on NB-IoT. Aquacult. Eng..

[bib70] Idachaba, Francis E., Olowoleni Joseph O., Ibhaze Augustus E., Oni Oluyinka O. (2017). IoT enabled real-time fishpond management system. Lect. Notes Eng. Comput. Sci..

[bib71] Jagtap Sandeep, Duong Linh Nguyen Khanh (2019). Improving the new product development using Big data: a case study of a food company. Br. Food J..

[bib72] Jagtap Sandeep, Duong Linh, Trollman Hana, Bader Farah, Garcia-garcia Guillermo, George Skouteris, Li Jie, Pathare Pankaj, Martindale Wayne, Swainson Mark, Rahimifard Shahin, Galanakis C.M. (2021). Food Technology Disruptions.

[bib73] Jagtap Sandeep, Garcia-Garcia Guillermo, Rahimifard Shahin (2021). Optimisation of the resource efficiency of food manufacturing via the Internet of Things. Comput. Ind..

[bib74] Janpla Satien, Tachpetpaiboon Nisanart, Jewpanich Chaiwat (2019). Development of automatic home-based fish farming using the Internet of Things. Int. J. Recent Technol. Eng..

[bib75] Kang Zhilong, Zhao Yuchen, Chen Lei, Guo Yanju, Mu Qingshuang, Wang Shenyi (2022). Advances in machine learning and hyperspectral imaging in the food supply chain. Food Eng. Rev..

[bib76] Karkal Sandesh S., Kudre Tanaji G. (2020). Valorization of fish discards for the sustainable production of renewable fuels. J. Clean. Prod..

[bib77] Karkal S.S., Kudre T.G. (2022). Valorization of marine fish waste biomass and Gallus Gallus eggshells as feedstock and catalyst for biodiesel production. Int. J. Environ. Sci. Technol..

[bib78] Kochanska Adrianna (2020).

[bib79] Kuswandi Bambang, Moradi Mehran, Ezati Parya (2022). Food sensors: off-package and on-package approaches. Packag. Technol. Sci..

[bib80] Kuuliala L., Pérez-Fernández R., Tang M., Vanderroost M., De Baets B., Devlieghere F. (2021). Probabilistic topic modelling in food spoilage analysis: a case study with atlantic Salmon (Salmo salar). Int. J. Food Microbiol..

[bib81] Kuzmina Ksenija, Prendeville Sharon, Walker Dale, Charnley Fiona (2019). Future scenarios for fast-moving consumer goods in a circular economy. Futures.

[bib82] Lekkas Demetris F., Panagiotakis Iraklis, Dermatas Dimitris (2021). A digital circular bioeconomy – opportunities and challenges for waste management in this new era. Waste Manag. Res..

[bib83] Li Pei, Ren Zouhong, Shao Kaiyi, Tan Hequn, Niu Zhiyou (2019). Research on distinguishing fish meal quality using different characteristic parameters based on electronic nose technology. Sensors.

[bib84] Li Nan, Ma Lianbo, Guo Yu, Xue Bing, Zhang Mengjie, Jin Yaochu (2022). Survey on evolutionary deep learning: principles, algorithms, applications and open issues. Neural Evol. Comput..

[bib85] Li Hongyue, Wang Yang, Zhang Jiaxin, Li Xuepeng, Wang Jinxiang, Yi Shumin, Zhu Wenhui, Xu Yongxia, Li Jianrong (2023). Prediction of the freshness of horse mackerel (Trachurus japonicus) using E-nose, E-tongue, and colorimeter based on biochemical indexes analyzed during frozen storage of whole fish. Food Chem..

[bib86] Liegeard Julie, Manning Louise (2020). Use of intelligent applications to reduce household food waste. Crit. Rev. Food Sci. Nutr..

[bib87] Lin Xianfeng, Han Yan, Zhao Lehan, Duan Nuo, Wang Zhouping, Wu Shijia (2023). Hydrogel-integrated sensors for food safety and quality monitoring: fabrication strategies and emerging applications. Crit. Rev. Food Sci. Nutr..

[bib88] Liu Xuwei, Le Carine, Yu Jiahao, Zhao Lei, Wang Kai, Yang Tao, Catherine M., Renard G.C., Hu Zhuoyan (2022). Trends and challenges on fruit and vegetable processing: insights into sustainable, traceable, precise, healthy, intelligent, personalized and local innovative food products. Trends Food Sci. Technol..

[bib89] Liu Yao, Xu Lele, Zeng Shaogeng, Qiao Fu, Jiang Wei, Xu Zhen (2022). Rapid detection of mussels contaminated by heavy metals using near-infrared reflectance spectroscopy and a constrained difference extreme learning machine. Spectrochim. Acta Mol. Biomol. Spectrosc..

[bib90] Lv Riqin, Huang Xingyi, Ye Weitao, Harrington Aheto Joshua, Xu Haixia, Dai Chunxia, Tian Xiaoyu (2019). Research on the reaction mechanism of colorimetric sensor array with characteristic volatile gases-TMA during fish storage. J. Food Process. Eng..

[bib91] Ma Ji, Sun Da-Wen, Qu Jia-Huan, Pu Hongbin (2017). Prediction of textural changes in grass carp fillets as affected by vacuum freeze drying using hyperspectral imaging based on integrated group wavelengths. LWT - Food Sci. Technol. (Lebensmittel-Wissenschaft -Technol.).

[bib92] Ma Peihua, Zhang Zhi, Xu Wenhao, Teng Zi, Luo Yaguang, Gong Cheng, Wang Qin (2021). Integrated portable shrimp-freshness prediction platform based on ice-templated metal–organic Framework colorimetric combinatorics and deep convolutional neural networks. ACS Sustain. Chem. Eng..

[bib93] Magnaghi Lisa Rita, Federica Capone, Zanoni Camilla, Alberti Giancarla, Quadrelli Paolo, Biesuz Raffaela (2020). Colorimetric sensor array for monitoring, modelling and comparing spoilage processes of different meat and fish foods. Foods.

[bib94] Majdinasab Marjan, Hosseini Seyed Mohammad Hashem, Sepidname Marziyeh, Negahdarifar Manizheh, Li Peiwu (2018). Development of a novel colorimetric sensor based on alginate beads for monitoring rainbow trout spoilage. J. Food Sci. Technol..

[bib95] Mao Xiangzhao, Guo Na, Sun Jianan, Xue Changhu (2017). Comprehensive utilization of shrimp waste based on biotechnological methods: a review. J. Clean. Prod..

[bib96] Meira Diana I., Barbosa Ana I., Joel Borges, Reis Rui L., Correlo Vitor M., Vaz Filipe (2023). Recent advances in nanomaterial-based optical biosensors for food safety applications: ochratoxin-A detection, as case study. Crit. Rev. Food Sci. Nutr..

[bib97] Mekki-Berrada Flore, Ren Zekun, Tan Huang, Wong Wai Kuan, Zheng Fang, Xie Jiaxun, Tian Isaac Parker Siyu, Jayavelu Senthilnath, Mahfoud Zackaria, Bash Daniil, Hippalgaonkar Kedar, Khan Saif, Buonassisi Tonio, Li Qianxiao, Wang Xiaonan (2021). Two-step machine learning enables optimized nanoparticle synthesis. npj Comput. Mater..

[bib98] Michelini Laura, Principato Ludovica, Iasevoli Gennaro (2018). Understanding food sharing models to tackle sustainability challenges. Ecol. Econ..

[bib99] Miguéis Vera Lucia, Pereira André, Pereira João, Figueira Gonçalo (2022). Reducing fresh fish waste while ensuring availability: demand forecast using censored data and machine learning. J. Clean. Prod..

[bib100] Moon Eui Jung, Kim Youngsik, Xu Yu, Yeul Na, Giaccia Amato J., Lee Jae Hyung (2020). Evaluation of salmon, tuna, and beef freshness using a portable spectrometer. Sensors.

[bib101] Mu B., Dong Y., Qian J., Wang M., Yang Y., Nikitina M.A., Zhang L., Xiao X. (2022). Hydrogel coating flexible PH sensor system for fish spoilage monitoring. Mater. Today Chem..

[bib102] Murali S., Soumya Krishnan V., Amulya P.R., Alfiya P.V., Delfiya D. S. Aniesran, Samuel Manoj P. (2021). Energy and water consumption pattern in seafood processing industries and its optimization methodologies. Clean. Eng. Technol..

[bib103] Muslihi Muhammad Takdir, Dani Achmad Andini (2019). Proceedings of the First International Conference on Materials Engineering and Management - Engineering Section.

[bib104] Mutalipassi Mirko, Esposito Roberta, Ruocco Nadia, Viel Thomas, Costantini Maria, Zupo Valerio (2021). Bioactive compounds of nutraceutical value from fishery and aquaculture discards. Foods.

[bib105] Nag Moupriya, Lahiri Dibyajit, Dey Ankita, Sarkar Tanmay, Pati Siddhartha, Joshi Sanket, Bunawan Hamidun, Mohammed Arifullah, Edinur Hisham Atan, Ghosh Sreejita, Ray Rina Rani (2022). Seafood discards: a potent source of enzymes and biomacromolecules with nutritional and nutraceutical significance. Front. Nutr..

[bib106] Natarajan Sowmya, Ponnusamy Vijayakumar (2022). Lecture Notes in Networks and Systems.

[bib107] Nayyar Sarita, de Cleene Sean, Dreier Lisa (2018).

[bib108] Ndraha Nodali, Hsiao Hsin I. (2022). A climate-driven model for predicting the level of Vibrio parahaemolyticus in oysters harvested from Taiwanese farms using elastic net regularized regression. Microb. Risk Anal..

[bib109] Ng Irene C.L., Wakenshaw Susan Y.L. (2017). The internet-of-things: review and research directions. Int. J. Res. Market..

[bib110] Nieto-Ortega Sonia, Olabarrieta Idoia, Saitua Eduardo, Gorka Arana, Foti Giuseppe, Melado-Herreros Ángela (2022). Improvement of oil valorization extracted from fish by-products using a handheld near infrared spectrometer coupled with chemometrics. Foods.

[bib111] Nirmal Nilesh Prakash, Maqsood Sajid (2022). Editorial: seafood waste utilization: isolation, characterization, functional and bio-active properties, and their application in food and nutrition. Front. Nutr..

[bib112] Nirmal, Prakash Nilesh, Santivarangkna Chalat, Singh Rajput Mithun, Benjakul Soottawat (2020). Trends in shrimp processing waste utilization: an industrial prospective. Trends Food Sci. Technol..

[bib113] Nirmal Nilesh P., Santivarangkna Chalat, Benjakul Soottawat, Maqsood Sajid (2022). Fish protein hydrolysates as a health-promoting ingredient—recent update. Nutr. Rev..

[bib114] Nirmal Nilesh Prakash, Santivarangkna Chalat, Singh Rajput Mithun, Benjakul Soottawat, Maqsood Sajid (2022). Valorization of fish byproducts: sources to end-product applications of bioactive protein hydrolysate. Compr. Rev. Food Sci. Food Saf..

[bib115] Nirmal Nilesh Prakash, Santivarangkna Chalat, Singh Rajput Mithun, Benjakul Soottawat, Maqsood Sajid (2022). Valorization of fish byproducts: sources to end‐product applications of bioactive protein hydrolysate. Compr. Rev. Food Sci. Food Saf..

[bib116] OECD/FAO (2022).

[bib117] Ogresta Lovro, Nekvapil Fran, Tǎmaş Tudor, Barbu-Tudoran Lucian, Suciu Maria, Hirian Rǎzvan, Aluaş Mihaela, Lazar Geza, Levei Erika, Glamuzina Branko, Pinzaru Simona Cintǎ (2021). Rapid and application-tailored assessment tool for biogenic powders from Crustacean shell waste: fourier transform-infrared spectroscopy complemented with X-ray diffraction, scanning electron microscopy, and nuclear magnetic resonance spectroscopy. ACS Omega.

[bib118] Olsen Ragnar L., Toppe Jogeir, Karunasagar Iddya (2014). Challenges and realistic opportunities in the use of by-products from processing of fish and shellfish. Trends Food Sci. Technol..

[bib119] Onwude Daniel I., Chen Guangnan, Eke-Emezie Nnanna, Abraham Kabutey, Khaled Alfadhl Yahya, Sturm Barbara (2020). Recent advances in reducing food losses in the supply chain of fresh agricultural produce. Processes.

[bib120] Oveissi Farshad, Nguyen Long H., Giaretta Jacopo E., Shahrbabaki Zahra, Rath Ronil J., Apalangya Vitus A., Yun Jimmy, Dehghani Fariba, Naficy Sina, Pablo Juliano R.B., Jay Sellahewa, Knoerzer Kai, Nguyen Minh (2022). Food Engineering Innovations across the Food Supply Chain.

[bib121] Owen Stephanie, Cureton Samuel, Szuhan Mathew, Joel McCarten, Arvanitis Panagiota, Ascione Max, Truong Vi Khanh, Chapman James, Cozzolino Daniel (2021). Microplastic adulteration in homogenized fish and seafood - a mid-infrared and machine learning proof of concept. Spectrochim. Acta Mol. Biomol. Spectrosc..

[bib122] Ozogul Fatih, Cagalj Martina, Šimat Vida, Ozogul Yesim, Tkaczewska Joanna, Hassoun Abdo, Kaddour Abderrahmane Ait, Kuley Esmeray, Rathod Nikheel Bhojraj, Phadke Girija Gajanan (2021). Recent developments in valorisation of bioactive ingredients in discard/seafood processing by-products. Trends Food Sci. Technol..

[bib123] Peñarubia Omar (2021). https://www.fao.org/flw-in-fish-value-chains/resources/articles/fish-by-products-utilization-getting-more-benefits-from-fish-processing/en/.

[bib125] Prapti Dipika Roy, Shariff Abdul Rashid Mohamed, Che Man Hasfalina, Ramli Norulhuda Mohamed, Perumal Thinagaran, Mohamed Shariff (2022). Internet of Things (IoT)-Based aquaculture: an overview of IoT application on water quality monitoring. Rev. Aquacult..

[bib126] Rashidi Shahnaz, Moghaddam Amin Hedayati (2021). Investigation and optimization of anaerobic system for treatment of seafood processing wastewater. Chem. Pap..

[bib127] Raveschot Cyril, Benoit Cudennec, Coutte François, Flahaut Christophe, Fremont Marc, Djamel Drider, Pascal Dhulster (2018). Production of bioactive peptides by lactobacillus species: from gene to application. Front. Microbiol..

[bib128] Rejeb Abderahman, Rejeb Karim, Zailani Suhaiza (2021). Big data for sustainable agri‐food supply chains: a review and future research perspectives. J. Digit. Inf. Manag..

[bib129] Rejeb Abderahman, Keogh John G., Rejeb Karim (2022). Big data in the food supply chain: a literature review. J. Digit. Inf. Manag..

[bib130] Riani Marco, Corbellini Aldo, Atkinson Anthony C. (2018). The use of prior information in very robust regression for fraud detection. Int. Stat. Rev..

[bib131] Roobab Ume, Fidalgo Liliana G., Arshad Rai Naveed, Khan Abdul Waheed, Zeng Xin An, Bhat Zuhaib F., Ala El Din A., Bekhit, Batool Zahra, Aadil Rana Muhammad (2022). High-pressure processing of fish and shellfish products: safety, quality, and research prospects. Compr. Rev. Food Sci. Food Saf..

[bib132] Rowan Neil J. (2022). The role of digital technologies in supporting and improving fishery and aquaculture across the supply chain – quo vadis?. Aquacult. Fish..

[bib133] Saha Sajal, Hasan Rajib Rakibul, Kabir Sumaiya (2018). 2018 International Conference on Innovations in Science, Engineering and Technology.

[bib134] Santos Vanessa P., Nathália S.S., Marques, Patrícia C.S., Maia V., Marcos Antonio Barbosa de Lima, de Oliveira Franco Luciana, de Campos-Takaki Galba Maria (2020). Seafood waste as attractive source of chitin and chitosan production and their applications. Int. J. Mol. Sci..

[bib135] Shahidi Fereidoon, Varatharajan Vamadevan, Han Peng, Senadheera Ruchira (2019). Utilization of marine by-products for the recovery of value-added products. J. Food Bioactives.

[bib136] Sharma Anshul, Sharma Rakesh K., Kim Yeon Kye, Lee Hae Jeung, Tripathi Kumud Malika (2021). Upgrading of seafood waste as a carbon source: nano-world outlook. J. Environ. Chem. Eng..

[bib137] Sharma Poonam, Vimal Archana, Vishvakarma Reena, Kumar Pradeep, porto de Souza Vandenberghe Luciana, Kumar Gaur Vivek, Varjani Sunita (2022). Deciphering the blackbox of omics approaches and artificial intelligence in food waste transformation and mitigation. Int. J. Food Microbiol..

[bib138] Sharma Selva, Arumugam, Marimuthu Murugavelu, Varghese Amal Wilson, Wu Jizong, Xu Jing, Luo Xiaofeng, Devaraj Sabarinathan, Yang Lan, Li Huanhuan, Chen Quansheng (2023). A review of biomolecules conjugated lanthanide up-conversion nanoparticles-based fluorescence probes in food safety and quality monitoring applications. Crit. Rev. Food Sci. Nutr..

[bib139] Singh Shikhangi, Negi Taru, Sagar Narashans Alok, Kumar Yogesh, Tarafdar Ayon, Sirohi Ranjna, Sindhu Raveendran, Pandey Ashok (2022). Sustainable processes for treatment and management of seafood solid waste. Sci. Total Environ..

[bib140] Singh Harminder, Sheetal Anu, Singh Maninder, Kaur Jaspreet, Tan Sui, Loja M.A.R., Trdan Uroš, Sharma Manupriya (2023). Electrical energy generation using fish scale of rohu fish by harvesting human motion mechanical energy for self powered battery-less devices. Sensor Actuator Phys..

[bib141] Skjelvareid Martin H., Heia Karsten, Olsen Stein Harris, Stormo Svein Kristian (2017). Detection of blood in fish muscle by constrained spectral unmixing of hyperspectral images. J. Food Eng..

[bib142] Suparmaniam Uganeeswary, Shaik Nagoor Basha, Lam Man Kee, Lim Jun Wei, Uemura Yoshimitsu, Shuit Siew Hoong, Show Pau Loke, Tan Inn Shi, Lee Keat Teong (2022). Valorization of fish bone waste as novel bioflocculant for rapid microalgae harvesting: experimental evaluation and modelling using back propagation artificial neural network. J. Water Proc. Eng..

[bib143] Suryawanshi Nisha, Satya Eswari J. (2022). Chitin from seafood waste: particle swarm optimization and neural network study for the improved chitinase production. J. Chem. Technol. Biotechnol..

[bib144] Swain Krishna Kumari, Balasubramaniam R., Bhand Sunil (2020). A portable microfluidic device-based Fe 3 O 4 –urease nanoprobe-enhanced colorimetric sensor for the detection of heavy metals in fish tissue. Prep. Biochem. Biotechnol..

[bib145] Tabrizi Seyfollah, Ghodsypour Seyed Hassan, Ahmadi Abbas (2018). Modelling three-echelon warm-water fish supply chain: a Bi-level optimization approach under nash–cournot equilibrium. Appl. Soft Comput..

[bib146] Talla Anuja, Stephen McIlwaine (2022). Industry 4.0 and the circular economy: using design-stage digital technology to reduce construction waste. Smart Sustain. Built. Environ. press.

[bib147] Tan, Wei Hsiao, Joan Lim Zhi Yin, Muhamad Nur Airina, Liew Fong Fong (2021). Potential economic value of chitin and its derivatives as major biomaterials of seafood waste, with particular reference to southeast asia. J. Renew. Mater..

[bib148] Teymouri Zahra, Shekarchizadeh Hajar (2022). A colorimetric indicator based on copper nanoparticles for volatile sulfur compounds to monitor fish spoilage in intelligent packaging. Food Packag. Shelf Life.

[bib149] Tian Chuang, Luan Weixin, Li Shijie, Xue Yunan, Jin Xiaoming (2021). Spatial imbalance of Chinese seafood restaurants and its relationship with socioeconomic factors. Ocean Coast Manag..

[bib150] Tichoniuk Mariusz, Bieganska Marta, Cierpiszewski Ryszard, Galanakis Charis M. (2021). Sustainable Food Processing and Engineering Challenges.

[bib151] Tseng, Lang Ming, Tran Thi Phuong Thuy, Ha Hien Minh, Bui Tat Dat, Ming K., Lim (2022). Causality of circular business strategy under uncertainty: a zero-waste practices approach in seafood processing industry in vietnam. Resour. Conserv. Recycl..

[bib152] Uhlmann Sven Sebastian, Ulrich Clara, Kennelly Steven J. (2019).

[bib153] UNEP DTU Partnership and United Nations Environment, and Programme (2021).

[bib154] Vajdi Meisam, Varidi Mohammad J., Varidi Mehdi, Mohebbi Mohebbat (2019). Using electronic nose to recognize fish spoilage with an optimum classifier. J. Food Meas. Char..

[bib155] Valdez Marisol, Gupta Santosh K., Lozano Karen, Mao Yuanbing (2019). ForceSpun polydiacetylene nanofibers as colorimetric sensor for food spoilage detection. Sensor. Actuator. B Chem..

[bib156] Venugopal Vazhiyil (2009). Vazhiyil Venugopal.

[bib157] Venugopal V. (2021). Valorization of seafood processing discards: bioconversion and bio-refinery approaches. Front. Sustain. Food Syst..

[bib158] Venugopal Vazhiyil (2022). Green processing of seafood waste biomass towards blue economy. Current. Res. Environ. Sustain..

[bib159] Venugopal Vazhiyil, Sasidharan Abhilash (2022). Functional proteins through green refining of seafood side streams. Front. Nutr..

[bib160] Visconti Paolo, de Fazio Roberto, Velázquez Ramiro, Del-Valle-soto Carolina, Giannoccaro Nicola Ivan (2020). Development of sensors-based agri-food traceability system remotely managed by a software platform for optimized farm management. Sensors.

[bib161] Wang Dongyi, Vinson Robert, Holmes Maxwell, Seibel Gary (2018). Convolutional neural network guided blue crab knuckle detection for autonomous crab meat picking machine. Opt. Eng..

[bib162] Wu Yuanhong, Zhuang Rui, Cui Zhendong (2019). 2019 International Conference on High Performance Big Data and Intelligent Systems.

[bib163] Xu Xia, Wu Xiaotian, Zhuang Shunqian, Zhang Yucong, Ding Yuting, Zhou Xuxia (2022). Colorimetric biosensor based on magnetic enzyme and gold nanorods for visual detection of fish freshness. Biosensors.

[bib164] Yadav Anamika, Noori Md Tabish, Biswas Abhijit, Min Booki (2022).

[bib165] Yang Fang, Guo Honghui, Gao Pei, Yu Dawei, Xu Yanshun, Jiang Qixing, Yu Peipei, Xia Wenshui (2021). Comparison of methodological proposal in sensory evaluation for Chinese mitten crab (eriocheir sinensis) by data mining and sensory panel. Food Chem..

[bib166] Yang Manyun, Luo Yaguang, Sharma Arnav, Jia Zhen, Wang Shilong, Wang Dayang, Lin Sophia, Perreault Whitney, Purohit Sonia, Gu Tingting, Dillow Hyden, Liu Xiaobo, Yu Hengyong, Zhang Boce (2022). Nondestructive and multiplex differentiation of pathogenic microorganisms from spoilage microflora on seafood using paper chromogenic array and neural network. Food Res. Int..

[bib167] Yue Kangning, Shen Yubang (2022). An overview of disruptive technologies for aquaculture. Aquacult. Fish..

[bib168] Zhang Yituo, Wang Xue, Shan Jiajia, Zhao Junbo, Zhang Wei, Liu Lifen, Wu Fengchang (2019). Hyperspectral imaging based method for rapid detection of microplastics in the intestinal tracts of fish. Environ. Sci. Technol..

[bib169] Zhang Yaqin, Luo Qian, Ding Ke, Liu Shi Gang, Shi Xingbo (2021). A smartphone-integrated colorimetric sensor of total volatile basic nitrogen (TVB-N) based on Au@MnO2 core-shell nanocomposites incorporated into hydrogel and its application in fish spoilage monitoring. Sens. Actuators, B.

[bib170] Zhang Nan, Lim Sheng Jie, Jia Min Toh, Wei Yue Fan, Rusli, Lin Ke (2022). Investigation of spoilage in salmon by electrochemical impedance spectroscopy and time-domain terahertz spectroscopy. Chem. Phys. Mater..

[bib171] Zhao Zezhong, Li Yajuan, Du Zhiyang (2022). Seafood waste-based materials for sustainable food packing: from waste to wealth. Sustainability.

[bib172] Zhou Xun, Qin A.K., Gong Maoguo, Tan Kay Chen (2021). A survey on evolutionary construction of deep neural networks. IEEE Trans. Evol. Comput..

[bib173] Zhu Jingyu, Luo Zhenyi, Liu Yuru, Tong Huanhuan, Yin Ke (2022). Environmental perspectives for food loss reduction via smart sensors: a global life cycle assessment. J. Clean. Prod..

